# Potential of traditional medicines in alleviating COVID-19 symptoms

**DOI:** 10.3389/fphar.2024.1452616

**Published:** 2024-09-26

**Authors:** Moragot Chatatikun, Hiroko P. Indo, Motoki Imai, Fumitaka Kawakami, Makoto Kubo, Yoshimasa Kitagawa, Hiroshi Ichikawa, Lunla Udomwech, Atthaphong Phongphithakchai, Orawan Sarakul, Suriyan Sukati, Voravuth Somsak, Takafumi Ichikawa, Wiyada Kwanhian Klangbud, Veeranoot Nissapatorn, Jitbanjong Tangpong, Hideyuki J. Majima

**Affiliations:** ^1^ School of Allied Health Sciences, Walailak University, Nakhon Si Thammarat, Thailand; ^2^ Center of Excellence Research for Melioidosis and Microorganisms, Walailak University, Nakhon Si Thammarat, Thailand; ^3^ Research Excellence Center for Innovation and Health Products (RECIHP), School of Allied Health Sciences, Walailak University, Nakhon Si Thammarat, Thailand; ^4^ Department of Oncology, Kagoshima University Graduate School of Medical and Dental Sciences, Kagoshima, Japan; ^5^ Amanogawa Galaxy Astronomy Research Center, Kagoshima University Graduate School of Engineering, Kagoshima, Japan; ^6^ Department of Regulation Biochemistry, Kitasato University Graduate School of Medical Sciences, Sagamihara, Japan; ^7^ Department of Health Administration, School of Allied Health Sciences, Kitasato University, Sagamihara, Japan; ^8^ Regenerative Medicine and Cell Design Research Facility, School of Allied Health Sciences, Kitasato University, Sagamihara, Japan; ^9^ Division of Microbiology, Kitasato University School of Allied Health Sciences, Sagamihara, Japan; ^10^ Department of Environmental Microbiology, Graduate School of Medical Sciences, Kitasato University, Sagamihara, Japan; ^11^ Oral Diagnosis and Medicine, Division of Oral Pathobiological Science, Graduate School of Dental Medicine, Hokkaido University, Sapporo, Japan; ^12^ Graduate School of Life and Medical Sciences, Doshisha University, Kyoto, Japan; ^13^ School of Medicine, Walailak University, Nakhon Si Thammarat, Thailand; ^14^ Division of Nephrology, Department of Internal Medicine, Faculty of Medicine, Prince of Songkla University, Songkhla, Thailand; ^15^ Medical Technology Program, Faculty of Science, Nakhon Phanom University, Nakhon Phanom, Thailand

**Keywords:** COVID-19, oxidative disease, immune reaction, immune evasion, traditional medicine, natural metabolites, botanical drugs, long covid

## Abstract

This review discusses the prevention and treatment of coronavirus disease 2019 (COVID-19) caused by infection with the severe acute respiratory syndrome coronavirus 2 (SARS-CoV-2). Mutations in its spike glycoprotein have driven the emergence of variants with high transmissibility and immune escape capabilities. Some antiviral drugs are ineffective against the BA.2 subvariant at the authorized dose. Recently, 150 natural metabolites have been identified as potential candidates for development of new anti-COVID-19 drugs with higher efficacy and lower toxicity than those of existing therapeutic agents. Botanical drug-derived bioactive molecules have shown promise in dampening the COVID-19 cytokine storm and thus preventing pulmonary fibrosis, as they exert a strong binding affinity for viral proteins and inhibit their activity. The Health Ministry of Thailand has approved *Andrographis paniculata* (Jap. Senshinren) extracts to treat COVID-19. In China, over 85% of patients infected with SARS-CoV-2 receive treatments based on traditional Chinese medicine. A comprehensive map of the stages and pathogenetic mechanisms related to the disease and effective natural products to treat and prevent COVID-19 are presented. Approximately 10% of patients with COVID-19 are affected by long COVID, and COVID-19 infection impairs mitochondrial DNA. As the number of agents to treat COVID-19 is limited, adjuvant botanical drug treatments including vitamin C and E supplementation may reduce COVID-19 symptoms and inhibit progression to long COVID.

## 1 Introduction

Coronavirus disease 2019 (COVID-19) is associated with oxidative stress (Beltrán-García et al., 2020; Esmaeili-Nadimi et al., 2023). In March 2003, a novel coronavirus was isolated from patients with atypical pneumonia and subsequently proven to be the causative agent of the disease now referred to as severe acute respiratory syndrome (SARS) (Lu et al., 2020). In late December 2019, COVID-19 was first reported in Wuhan, China. Lu et al. (2020) identified as the underlying cause a new human-infecting beta-coronavirus initially called 2019-nCoV which sufficiently differed from the severe acute respiratory syndrome coronavirus (SARS-CoV). Wu and McGoogan (2020) compared the time courses of the two SARS outbreaks in 2002–2003 and 2019–2020, showing that the two diseases followed a similar course of events, although the number of cases in China was relatively low during the SARS 2002–2003 outbreak (Wu et al., 2023). Subsequently, several variants of SARS-CoV-2, namely Alpha, Beta, Gamma (Lazarevic et al., 2021), Delta (Burki, 2021; Lazarevic et al., 2021; Noori et al., 2022), and Omicron (National Center for Immunization and Respiratory Diseases (NCIRD), Division of Viral Diseases, 2020), resulted in various outbreaks in many countries worldwide.

Evidently, SARS-CoV-2 continues to mutate (GISAID, 2021), and the current variant, Omicron BA.2, has spread worldwide. The genome of the Omicron variant is characterized by some deletions and more than 30 mutations, several of which (e.g., 69–70 del, T95I, G142D/143–145del, K417N, T478K, N501Y, N655Y, N679K, and P681H) overlap with those in the genomes of the Alpha, Beta, Gamma, or Delta variants (GISAID, 2021; Karim and Karim, 2021). These deletions and mutations led to increased transmissibility, higher viral binding affinity, and higher antibody escape (Greaney et al., 2021; Harvey et al., 2021; Karim and Karim, 2021). Escape mutations cluster on several surfaces of the receptor-binding domain, broadly corresponding to structurally defined antibody epitopes (Greaney et al., 2021; Harvey et al., 2021; Karim and Karim, 2021). The spike glycoprotein of SARS-CoV-2 has driven the emergence of variants with high transmissibility and immune escape capabilities (Souza et al., 2022). Antibodies targeting the spike receptor-binding domain of the virus are a major contributor to neutralizing antibody responses elicited by SARS-CoV-2 infection and are being developed as therapeutics (Min and Sun, 2021).

## 2 Clinical symptoms of COVID-19

**FIGURE 1:**
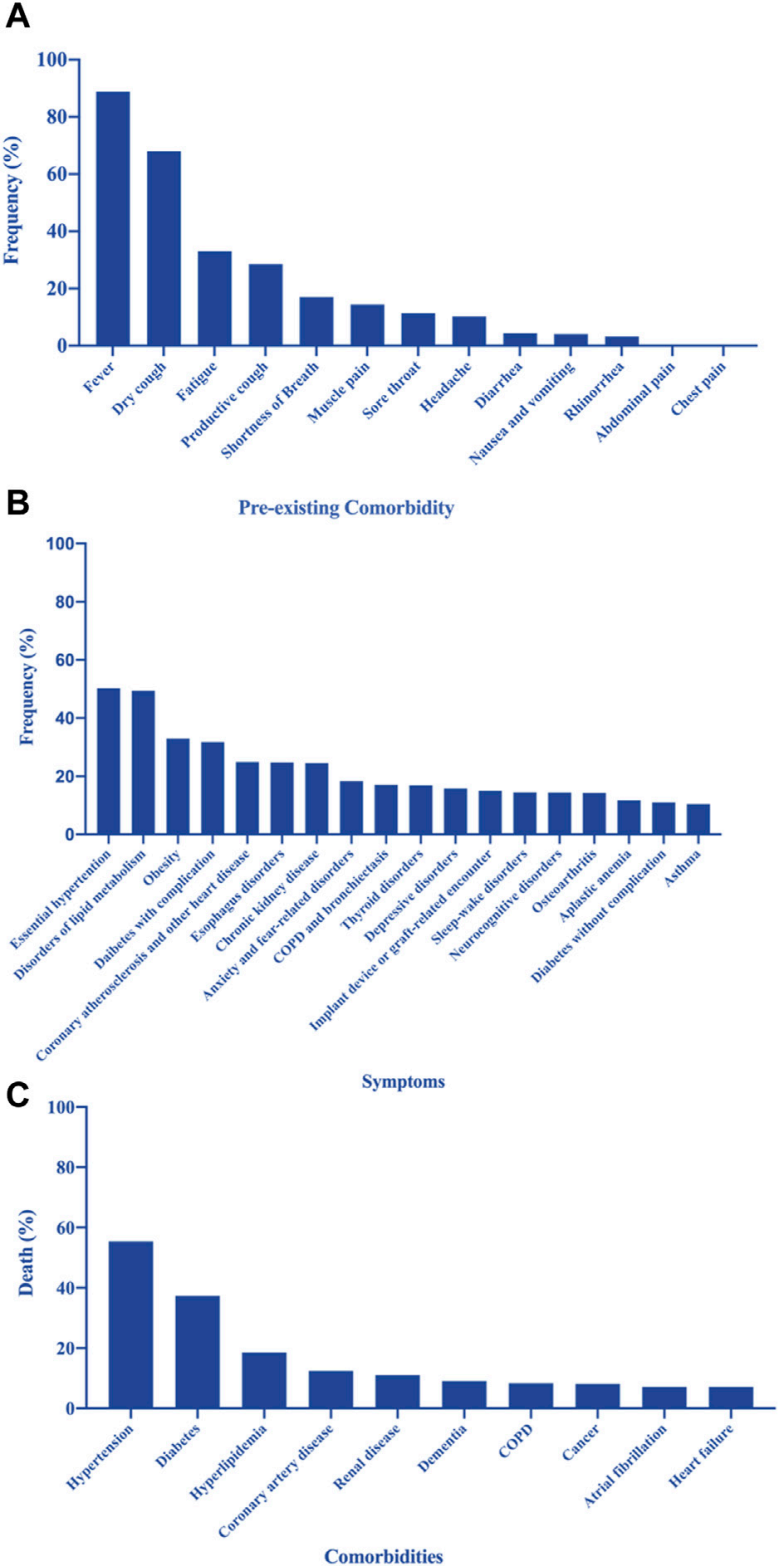
**(A)** Pre-existing comorbidities of patients with coronavirus disease 2019 (COVID-19) (Paudel, 2020; Sanyaolu et al., 2020). **(B)** Prevalence of the most frequent underlying medical conditions in a sample of adults hospitalized with COVID-19 (Kompaniyets et al., 2021). **(C)** Leading causes of death in patients with COVID-19 in New York, United States (Franki, 2020; Sanyaolu et al., 2020).

Various clinical symptoms of COVID-19 have been reported worldwide (WHO, 2023). Figure 1A shows the clinical characteristics of COVID-19 as reported by Paudel (2020) based on a cohort of 1,786 patients (1,044 men and 742 women; mean age, 41 years) between January 24 and 16 March 2020 (Sanyaolu et al., 2020). The most common symptoms observed in patients with COVID-19 were fever (89%), dry cough (68%), fatigue (33%), productive cough (29%), shortness of breath (17%), muscle pain (14%), sore throat (11%), headache (10%), diarrhea (4%), nausea and vomiting (4%), rhinorrhea (3%), abdominal pain (>1%), and chest pain (>1%). These findings suggest that the infection starts in the respiratory organs, subsequently spreading to the digestive system. It has been noted that olfactory and gustatory dysfunction may precede or accompany typical COVID-19 features, such as fever and cough (Horvath et al., 2021), and might persist long after the resolution of respiratory symptoms (Mastrangelo et al., 2021). Loss of taste was reported by 60% of patients. The time to the development of olfactory dysfunction was significantly shorter in patients with mild-to-moderate symptoms than in those with severe symptoms; in 85% of these patients, this symptom resolved within the first 2 months. However, 15% of patients remained symptomatic after 3 months of follow-up (Parente-Arias et al., 2021). Although most patients with COVID-19 recover quickly, nearly one out of ten do not recover within 2 months (Printza et al., 2021). These persistent symptoms constitute what has been termed “long COVID” (Makaronidis et al., 2021).

Figure 1B shows the prevalence of the most frequent underlying medical conditions in a population of hospitalized adults with COVID-19 infection (Kompaniyets et al., 2021). Figure 1C shows the leading comorbidities associated with COVID-19 deaths in New York, United States (Franki, 2020; Sanyaolu et al., 2020). Gupta et al. (2021) reported that asthma, history of cancer, coronary artery disease, chronic kidney disease without dialysis, end-stage renal disease on dialysis, diabetes mellitus, hyperlipidemia, and immune suppression cause a significant increase in mortality among hospitalized patients with COVID-19 (Gupta et al., 2021).

Corona et al. (2021) provided an overview and a meta-analysis of the main predictors and differences in COVID-19-associated mortality in hospitalized patients. Of 3,714 retrieved articles, 87 studies were considered, including 35,486 patients (mean age, 60.9 ± 8.2 years) and 5,867 deaths. All assessed co-morbidities were significantly correlated with higher mortality rates, even after adjusting for confounders. The strongest predictors of mortality were associated comorbidities, such as arterial hypertension, diabetes mellitus, chronic obstructive pulmonary disease, malignancies, and cardiovascular disease; clinical symptoms, such as dyspnea, elevated respiratory rate, and myalgia; and laboratory parameters, such as reduced lymphocyte count, reduced platelet count, and elevated D-dimer (Corona et al., 2021).

## 3 Treatment options for COVID-19

### 3.1 Approved medicines for COVID-19

#### 3.1.1 Ritonavir-boosted nirmatrelvir (Paxlovid), remdesivir, and molnupiravir

**FIGURE 2:**
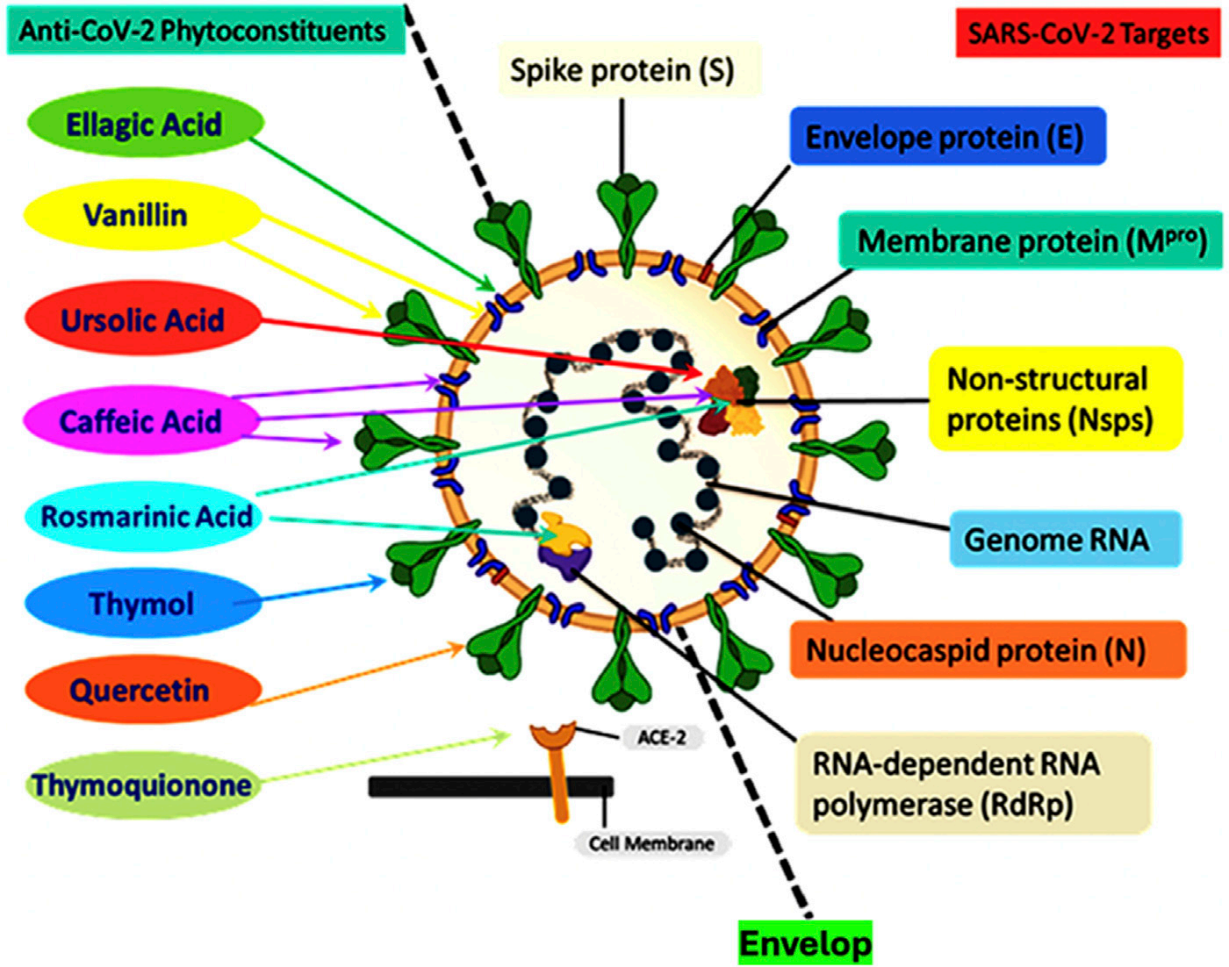
Schematic representation of the structure of the severe acute respiratory syndrome coronavirus 2 (SARS-CoV-2) showing the viral genome and important viral proteins: spike protein (S), nucleocapsid protein (N), envelope protein (E), membrane protein (M^pro^), non-structural proteins (Nsps), and RNA-dependent RNA polymerase (RdRp). Natural phytochemicals showed therapeutic potential against SARS-CoV-2 by binding to these proteins and inhibiting their functions. Phytochemicals such as thymoquinone interact with ACE2 receptors to block the virus entry; thymol interacts with the S protein; quercetin interacts with 3-chymotrypsin-like protease (3CL^pro^); vanillin interacts with both the 3CL^pro^ and S proteins; rosmarinic acid interacts with NSP-15; ursolic acid interacts with NSP-15 and M proteins; ellagic acid interacts with M proteins and caffeic acid, and its derivatives interact with M proteins, NSP-15, and the spike protein. Redrawn from (Ali et al., 2022).

Figure 2 shows a schematic representation of the SARS-CoV-2 structure including the viral genome and important viral proteins, such as the spike protein (S), nucleocapsid protein (N), envelope protein (E), membrane protein (M^pro^), non-structural proteins (Nsps), and RNA-dependent RNA polymerase (RdRp). Several natural phytochemicals show therapeutic potential against SARS-CoV-2 by binding to these proteins and inhibiting their functions (Ali et al., 2022).

The National Institutes of Health recommends ritonavir-boosted nirmatrelvir, remdesivir, and molnupiravir for non-hospitalized patients with COVID-19 (NIH, 2024). Paxlovid (Pfizer) is a co-packaged combination of nirmatrelvir and ritonavir tablets intended for co-administration and was developed for the treatment and post-exposure prophylaxis of COVID-19 (Lamb, 2022). It is used as an oral antiviral medicine for mild-to-moderate COVID-19 in patients aged ≥12 years, weighing at least 88 pounds (40 kg), who are at high risk of severe illness, including hospitalization or death (Anderson, 2023). Nirmatrelvir, an orally bioavailable protease inhibitor, inhibits SARS-CoV-2 replication by targeting the main viral protease M^pro^ (Hammond et al., 2022; Joyce et al., 2022). This protease plays an essential role in viral replication by cleaving two viral polyproteins (Pillaiyar et al., 2016). Ritonavir inhibits the nirmatrelvir-metabolizing enzyme cytochrome P450 3A to lengthen the half-life of nirmatrelvir, thus acting as a pharmacological enhancer (Hashemian et al., 2023). Ritonavir has previously been used to boost HIV protease inhibitors (Pillaiyar et al., 2016). Co-administration of ritonavir is required to raise the nirmatrelvir concentrations into the target therapeutic range (NIH, 2024). Nirmatrelvir plus ritonavir received its first conditional authorization in December 2021 in the United Kingdom for the treatment of COVID-19 in adults who do not require supplemental oxygen and who are at increased risk of progression to severe COVID-19. In January 2022, nirmatrelvir plus ritonavir received authorization in the EU for the same indication. Nirmatrelvir plus ritonavir is also authorized for emergency use in the United States (Lamb, 2022). These are recent FDA-approved drugs for the treatment of COVID-19 (Ghosh et al., 2022). Molnupiravir and remdesivir inhibit SARS-CoV-2 replication by targeting RdRp (Yan and Yan, 2023). Remdesivir, which is only used in hospitalized patients, is a nucleotide prodrug of an adenosine analog. It binds to the viral RdRp and inhibits viral replication by terminating RNA transcription prematurely (NIH, 2024). Remdesivir has demonstrated *in vitro* activity against SARS-CoV-2 (Frediansyah et al., 2021). Molnupiravir is the oral prodrug of β-D-N4-hydroxycytidine, a ribonucleoside that has shown antiviral activity against SARS-CoV-2 *in vitro* and in clinical trials (Arribas et al., 2022; Strizki et al., 2023). The uptake of β-D-N4-hydroxycytidine by the virus and its interaction with RdRps result in viral mutations and lethal mutagenesis (Zhou et al., 2021; Zibat et al., 2023).

#### 3.1.2 Adverse effects of approved medicines for COVID-19

Paxlovid is not recommended for COVID-19 patients with severe renal or hepatic impairment. Dose adjustment is required for patients with mild-to-moderate renal or hepatic impairment (Islam et al., 2022). By contrast, the doses of remdesivir and molnupiravir do not need adjustment in patients with renal or hepatic impairment (Islam et al., 2022). The unwanted effects of ritonavir-boosted nirmatrelvir, remdesivir, and molnupiravir are listed in Table 1.

**TABLE 1:** Adverse effects of medicines approved for COVID-19: nirmatrelvir (Paxlovid), remdesivir, and molnupiravir.

Drug	Adverse effects and remarks	References
Nirmatrelvir and ritonavir (Paxlovid)	Bitter or metallic taste, diarrhea, hypertension, nausea or vomiting, stomach pain.	Nguyen and Prouty (2023)
	Dysgeusia, diarrhea, hypertension, myalgia, severe allergies (anaphylaxis), liver problems, kidney problems, resistance to HIV medicines, drug interactions.	Anderson (2023)
	Changes in taste (“Paxlovid mouth”), diarrhea, hypertension, myalgia.	Aungst and Murdock (2023)
	Less common: blurred vision, dizziness, headache, nervousness, pounding in the ears, brady- or tachycardia. Incidence not known: blistering, peeling, or loosening of the skin; chest tightness; chills; clay-colored stools; cough; dark urine; diarrhea; difficulty swallowing; tachycardia; fever; unusual or general tiredness or weakness; hives, itching, or skin rash; joint or muscle pain; large, hive-like swelling on the face, eyelids, lips, tongue, throat, hands, legs, feet, or sex organs; light-colored stools; loss of appetite; nausea and vomiting; puffiness or swelling of the eyelids or around the eyes, face, lips, or tongue; red irritated eyes; red skin lesions, often with a purple center; sore throat; sores, ulcers, or white spots in the mouth or on the lips; halitosis; upper right abdominal or stomach pain; hematemesis; jaundice.	Drugs.com (2023)
	Common: changes in taste, diarrhea. Serious: liver problems, loss of appetite, stomach pain (upper right side), tiredness, itching, dark urine, clay-colored stools, jaundice.	Multum (2024)
	Common (Paxlovid): bad taste or change in taste sensation, diarrhea, headache, vomiting, stomach pain, nausea, hypertension. Serious (Paxlovid): allergic reactions (hives, difficulty breathing, swelling in the face or throat), severe skin reactions (fever, sore throat, burning eyes, skin pain, red or purple skin rash with blistering and peeling). Hepatic symptoms: loss of appetite, stomach pain (upper right side), tiredness, itching, dark urine, clay-colored stools, jaundice.	Puckey (2024)
Ritonavir	300 mg of nirmatrelvir plus 100 mg of ritonavir (a pharmacokinetic enhancer)	Hammond et al. (2022)
	Ritonavir affects the immune system. Adverse effects (even weeks or months after administration of nirmatrelvir and ritonavir): new infections, fever, night sweats, swollen glands, cold sores, cough, wheezing, diarrhea, weight loss, trouble speaking or swallowing, problems with balance or eye movement, weakness or prickly feeling, swelling in the neck or throat (enlarged thyroid), menstrual changes, impotence.	Puckey (2024)
Remdesivir	Most common: nausea. Hypersensitivity, including infusion-related anaphylactic reactions, has been observed following treatment. There is insufficient data on the safety of using remdesivir in women and pregnant women. The use of remdesivir was associated with an increased reporting of kidney disorders.	Yap (2023)
Molnupiravir (Lagevrio)	Effective only when used within 5 days of symptom onset.	Singh et al. (2022)
	Common: diarrhea, nausea, dizziness. Severe: potential allergic reactions, which may cause skin rash, hives, or severe swelling of the face or throat.	Nguyen and Prouty (2023)
	Diarrhea, nausea, dizziness.	Aungst and Murdock (2023)

### 3.2 Palliative treatment for COVID-19

#### 3.2.1 Selected medicines for COVID-19 treatment

The review by Kuchta et al. (2022) discussed which East Asian botanical drugs can decrease the viral load in infections (Kuchta et al., 2022). Park et al. (2012) introduced the history of traditional medicine in China, Korea, and Japan (Park et al., 2012). In East Asia, traditional botanical drug prescriptions are being studied for their use in SARS-CoV-2 therapy. In Japan, Maoto is used based on clinical evidence for its promising effects in the early phase of influenza (also caused by RNA viruses), comparable to those of oseltamivir. Maoto consists of four individual raw drugs: *Ephedrae herba* [Ephedraceae; *Herba Ephedrae*], *Armeniacae semen* [Rosaceae; *Semen Armeniacae*], *Cinnamomi cortex* [Lauraceae; *Cortex Cinnamomi*], and *Glycyrrhizae radix* [Fabaceae; *Radix Glycyrrhizae*]. Among these, *Ephedrae herba*, *C. cortex*, and *G. radix* have been identified as the most promising antiviral components compared with other Kampo prescriptions that have proven antiviral properties (Kuchta et al., 2022). Nabeshima et al. (2022) further suggested that oral administration of Maoto might be effective as post-exposure prophylaxis against nosocomial COVID-19 infection (Nabeshima et al., 2022).

In China, supportive care and non-specific treatments to alleviate symptoms are currently the only options available for managing COVID-19. Over 85% of SARS-CoV-2-infected patients in China receive treatment based on traditional Chinese medicine (Yang et al., 2020). Among the metabolites of candidate botanical drugs, 13 molecules bind to papain-like protease (PL^pro^) or 3-chymotrypsin-like protease (3CL^pro^), which are candidate targets for COVID-19 treatment (Yang et al., 2020; Malekmohammad and Rafieian-Kopaei, 2021). The official COVID-19 treatment guidelines in China recommend formulations of traditional Chinese medicine with antiviral, immunomodulatory, and anti-inflammatory properties. Examples include Qing-Fei-Pai-Du-Tang (containing 21 plants), Shufeng Jiedu Jiaonang (containing *Bupleuri radix* [Apiaceae; *Radix Bupleuri*], *Forsythiae fructus* [Oleaceae; *Fructus Forsythiae*], *G. radix* [Fabaceae; *Radix Glycyrrhizae*], *Isatidis radix* [Brassicaceae; *Radix Isatidis*], *Patriniae herba* [Caprifoliaceae; *Herba Patriniae*], *Phragmitis rhizome* [Poaceae; *Rhizoma Phragmitis*], *Polygoni cuspidati rhizome* [Polygonaceae; *Rhizoma Polygoni Cuspidati*], and *Verbenae herba* [Verbenaceae; *Herba Verbenae*]), and Fufang Yuxingcao Heiji (containing *Forsythiae fructus* [Oleaceae; *Fructus Forsythiae*], *Houttuyniae herba* [Saururaceae; *Herba Houttuyniae*], *Isatidis radix* [Brassicaceae; *Radix Isatidis*], *Lonicerae flos* [Caprifoliaceae; *Flos Lonicerae*], and *Scutellariae radix* [Lamiaceae; *Radix Scutellariae*]). These selected traditional medicines first gained prominence during the SARS epidemic in 2002 (Park et al., 2012).

To prevent COVID-19 and for palliative care of COVID-19 symptoms, many adjunctive therapies have been introduced in addition to the established medications. Currently, clinical trials using Kampo, a Japanese variant of traditional Chinese medicine, are being conducted (Takayama et al., 2021). In these trials, 78 types of Kampo botanical drugs were administered for the palliative treatment of COVID-19 (Takayama et al., 2021). Hydroxychloroquine is often prescribed and is partially effective (Klouda and Stone, 2020; Prodromos and Rumschlag, 2020). Since COVID-19 induces oxidative stress, Mironova et al. (2020) suggested that dihydroquercetin, an effective free radical scavenger, might be a promising agent for treating COVID-19 (Mironova et al., 2020).

The Health Ministry of Thailand has approved *Andrographis paniculata* (Jap. Senshinren) extracts to treat COVID-19. Its active ingredients are ephedrine, norephedrine, pseudoephedrine, D-norpseudoephedrine, L-methylephedrine, L-ephedrine, and D-pseudoephedrine (Kuchta et al., 2022). Recently, the introduction of *A*. *paniculata* into the Chinese pharmacopoeia and the current efforts to integrate more botanical drugs of East Asian Medicine into the European regulatory framework have led to its inclusion in the newest edition of the European pharmacopoeia. Currently, *A*. *paniculata* (Burm. f.) Nees [Acanthaceae; *Herba Andrographidis*] is widely used worldwide, especially in subtropical regions such as India, Thailand, Vietnam, and China (Jiang et al., 2021; Intharuksa et al., 2022).

#### 3.2.2 Additional zinc and proper nutrition

Insufficient or excess iron can lead to an increased risk of infection (Cavezzi et al., 2020; Edeas et al., 2020), whereas zinc is necessary for maintaining immune functions (Maares and Haase, 2016). Malnutrition is dangerous for patients with COVID-19; therefore, proper nutrition should be prioritized (Fernández-Quintela et al., 2020; Kurtz et al., 2021; van der Meij et al., 2021; Feng et al., 2022). In addition to diet, physical activity is another key factor. Individuals should be active and perform regular physical exercise to boost their immune systems and ensure sufficient sleep each night (Simpson et al., 2020; da Silveira et al., 2021).

## 4 Natural products and botanical drugs

### 4.1 Natural products and botanical drugs for the treatment of COVID-19

Natural products can interfere with COVID-19 pathogenesis by inhibiting SARS-CoV-2 replication and entry into host cells (Demeke et al., 2021). Islam et al. (2021) published a list of pharmacological drugs and natural bioproducts—natural bioactive molecules with varied biological significance and antiviral properties. They reviewed 40 different types of natural botanical drugs, including alkaloids, polyphenols, flavonoids, terpenoid derivatives, and other metabolites that might be used to treat COVID-19 (Islam et al., 2021). Mukherjee et al. (2022) also reviewed the use of medicinal plants for treatment and control of COVID-19. They summarized 625 articles from more than 18 countries and reported data collected from over 95 medicinal plants and 25 active phytomolecules belonging to 48 different plant families, which should be further explored to develop safe and efficacious products targeting COVID-19 (Mukherjee et al., 2022).

### 4.2 Potential targets of natural products in COVID-19 infection and treatment

COVID-19 infection involves multiple steps following the entry of the virus into host cells. The SARS-CoV-2 spike protein binds to angiotensin-converting enzyme 2 (ACE2) on host cells, followed by priming of the spike protein S by transmembrane protease serine 2 (TMPRSS2). The virus produces the polyproteins pp1a and pp1ab, which are processed by viral proteases (3CL^pro^, M^pro^, and PL^pro^) to non-structural proteins, including RdRp (Lan et al., 2020; Yang et al., 2020; Jackson et al., 2022). Viral RdRp synthesizes a full-length complementary negative-strand RNA as a template to produce the positive-strand genome of the virus. Sub-genomic mRNAs are then translated into structural proteins in the rough endoplasmic reticulum or the cytosol (Nicholson and White, 2014). The viral genomic RNA is encapsulated by the nucleocapsid protein N, and finally, the virus is released by exocytosis (Cubuk et al., 2021; Wu et al., 2023).

Prasansuklab et al. (2021) described potential therapeutic mechanisms targeting individual replication steps and grouped them into four main categories based on the cellular and molecular machinery required for the viral reproductive cycle and related pathogenic mechanisms: 1) Inhibition of virus entry: The selective blockade of the spike protein binding to ACE2 and inhibition of TMPRSS2 activity would interfere with virus entry into the host cells. 2) Inhibition of viral replication: Inhibition of endocytic pathway-associated proteins, such as clathrin, vacuolar-type H^+^-ATPase, and cathepsin L, can potentially block viral replication inside the host cells. 3) Blocking the release of viral progenies: Viral multiplication can be blocked through direct inhibition of the proteolytic activity of two viral proteases, 3CL^pro^ and PL^pro^, and replicative activity of viral replication–transcription complex components, e.g., RdRp and helicase (Prasansuklab et al., 2021).

Each intracellular component of SARS-CoV-2 can be a therapeutic target in patients with COVID-19. Malekmohammad and Rafieian-Kopaei (2021) have described promising anti-SARS-CoV-2 actions of natural products. Different plant extracts, metabolites, and purified molecules may exert anti-SARS-CoV-2 activity by directly inhibiting the entry or replication of the virus. Some metabolites can block the ACE2 receptor or the serine protease TMPRSS2 required by SARS-CoV-2 to infect human cells. Other natural products inhibit SARS-CoV-2 life cycle-related proteins, such as PL^pro^ and 3CL^pro^ (Malekmohammad and Rafieian-Kopaei, 2021).

Using *in silico* studies, Chakravarti et al. (2021) have shown that various natural metabolites have strong binding affinity for and inhibitory action on Nsps of the virus, PL^pro^, M^pro^, and RdRp, as well as structural proteins, such as the spike protein. Since the virus utilizes the transmembrane ACE2 receptor of the host cell, it also seems to be a valid target for drug development (Chakravarti et al., 2021). Fan et al. (2021) have summarized the characteristics along with *in vitro* and *in vivo* activities of seven natural anti-complement metabolites for the treatment of severe COVID-19 (Fan et al., 2021). Singla et al. (2021) summarized *in silico* studies on 70 different phytochemicals and 10 botanical drug formulations effective against SARS-CoV-2. Multiple botanical drugs are efficacious against respiratory viral infections and can improve the treatment of COVID-19. A shortlist of botanical drug-derived bioactive molecules that reportedly inhibit viral entry, viral replication, and progression of COVID-19 include thymoquinone, α-hederin, nigellidine from *Nigella sativa* [Ranunculaceae; *Semen Nigellae*], quercetin found in *Ginkgo biloba* [Ginkgoaceae; *Folium Ginkgo*], green tea (*Camellia sinensis* [Theaceae; *Folium Camelliae*]), ellagic acid from *Moringa oleifera* [Moringaceae; *Folium Moringae*], and rosmarinic acid from *Plectranthus amboinicus* [Lamiaceae; *Herba Plectranthi*] (Singla et al., 2021). *Panax ginseng* [Araliaceae; *Radix Ginseng*] has shown therapeutic effects against COVID-19-related thrombosis and platelet aggregation. Curcumin, luteolin, piperine, quercetin, epigallocatechin-3-gallate, resveratrol, and botanical drugs of *Polygonum cuspidatum* [Polygonaceae; *Radix Polygoni Cuspidati*] exhibit the ability to dampen the COVID-19-related cytokine storm and thus prevent pulmonary fibrosis. Some medicinal fungi, such as extracts of *Ganoderma lucidum* [Ganodermataceae; *Fructus Ganodermae*], were capable of preventing SARS-CoV-2 infection in animal models (Isidoro et al., 2022). Comprehensive collections of studies on natural metabolites showing promising activity against human coronaviruses are freely accessible (Xian et al., 2020; Isidoro et al., 2022). Isidoro et al. (2022) discussed the relevance of an integrated approach that includes the tools and methodology of traditional and complementary medicine for managing COVID-19 (Isidoro et al., 2022).

*A*. *paniculata* (Burm. f.) Nees has antiviral activity (Kuchta et al., 2022). In traditional Thai medicine, *A. paniculata* (Fha Talai Jone) is a frequently used botanical drug (Singla et al., 2021; Kuchta et al., 2022), often administered as a decoction or pill to prevent a variety of health problems such as fever, cough, sore throat, aphthous ulcers, wounds, abscesses, rashes, gastritis, pain, diabetes, hypertension, and jaundice, as well as a carminative (Kuchta et al., 2022).

Nine guidelines provide botanical drug formulations for medical observation based on their clinical manifestation (Ang et al., 2020; Demeke et al., 2021), including 12 herbal formulations with 53 botanical drugs. Among those, *Citri reticulatae pericarpium* (Chenpi) [Rutaceae; *Pericarpium Citri Reticulatae*] and *G. radix et rhizome* (Gan Cao) [Fabaceae; *Radix et Rhizoma Glycyrrhizae*] are the most frequently used botanical drugs (Ang et al., 2020). The use of these botanical drugs may serve as a reference for future studies assessing COVID-19 treatment approaches (Ang et al., 2020; Demeke et al., 2021). Feng et al. (2022) reviewed beneficial botanical drugs and nutrients for COVID-19 prevention and recovery and identified some promising candidates for further studies. These candidates have few unwanted effects and might be evaluated to improve immune defense or recovery from COVID-19 (Feng et al., 2022). These authors listed adaptogens such as *Rhodiola rosea* [Crassulaceae; *Radix Rhodiolae*], *Eleutherococcus senticosus* [Araliaceae; *Radix et Rhizoma Eleutherococci*], *Schisandra chinensis* [Schisandraceae; *Fructus Schisandrae*], red mangrove of Fiji (*Rhizophora mangle*) [Rhizophoraceae; *Cortex Rhizophorae*], black elderberry (*Sambucus nigra*) [Adoxaceae; *Fructus Sambuci*], beta-glucan from mushrooms, olive leaf extract (*Olea europaea*) [Oleaceae; *Folium Oleae*], green chiretta (*A*. *paniculate* (Burm. f.) Nees) [Acanthaceae; *Herba Andrographidis*], French oak wood extract (*Quercus robur* extract) [Fagaceae; *Cortex Quercus*], and zinc. These botanical drugs and nutrients alleviate symptoms such as dyspnea, decreased respiratory rate, fatigue, and myalgia.

Based on *in silico* binding studies, Mosquera-Yuqui et al. (2022) showed that both hesperidin and lupinifolin are potent 3CL^pro^ inhibitors, suggesting their potential as COVID-19 treatments (Mosquera-Yuqui et al., 2022). Wen et al. (2007) suggested that terpenoids and lignoids possess potent antiviral activities against SARS-CoV-2 (Wen et al., 2007). Based on *in silico* binding simulations with 3CL^pro^, Tahir Ul Qamar et al. (2020) identified nine phytochemicals from various plants, including *Psorothamnus arborescens* [Fabaceae; *Herba Psorothamni*], *Myrica cerifera* [Myricaceae; *Cortex Myricae*], *Hyptis atrorubens* Poit [Lamiaceae; *Herba Hyptidis*], *Phaseolus vulgaris* [Fabaceae; *Semen Phaseoli*], *Phyllanthus emblica* [Phyllanthaceae; *Fructus Phyllanthi*], *Fraxinus sieboldiana* [Oleaceae; *Cortex Fraxini*], *C. sinensis* [Theaceae; *Folium Camelliae*], *Glycyrrhiza uralensis* [Fabaceae; *Radix Glycyrrhizae*], and *Amaranthus tricolor* [Amaranthaceae; *Herba Amaranthi*], as potential candidates for therapeutically targeting SARS-CoV-2 (Tahir Ul Qamar et al., 2020). Soleymani et al. (2022) described the efficacy of botanical drugs in the context of COVID-19, providing a comprehensive map of the stages and pathogenetic mechanisms related to the disease and effective natural products to treat and prevent COVID-19 (Soleymani et al., 2022). Ang et al. (2022) suggested that botanical drugs added to standard treatments have potential benefits in treating COVID-19 symptoms, but the certainty of their evidence is low (Ang et al., 2022).

### 4.3 Vitamin C and vitamin E supplementation

COVID-19 is a disease targeting mitochondria and leading to increased oxidative stress (Noonong et al., 2023). Mitochondria generate reactive oxygen species (ROS) and induce apoptosis (Majima et al., 1998). Mitochondrial DNA encodes 13 proteins out of a total of 100 proteins in the electron transfer chain, whose main role is to produce ATP using oxygen. Without oxygen, one glucose molecule produces 2 ATP molecules, whereas over 30 ATP molecules are produced in mitochondria (Indo et al., 2015). After SARS-CoV-2 has been taken up by a human cell, the virus enters the mitochondria, leading to excess ROS production and possibly destroying mitochondrial DNA. This can impair the ATP production in mitochondria (Noonong et al., 2023). Vitamin C and E supplementation might suppress excess ROS generation caused by the presence of SARS-CoV-2 in mitochondria (Majima et al., 2011; Kawashima et al., 2015).

## 5 Conclusion

**FIGURE 3:**
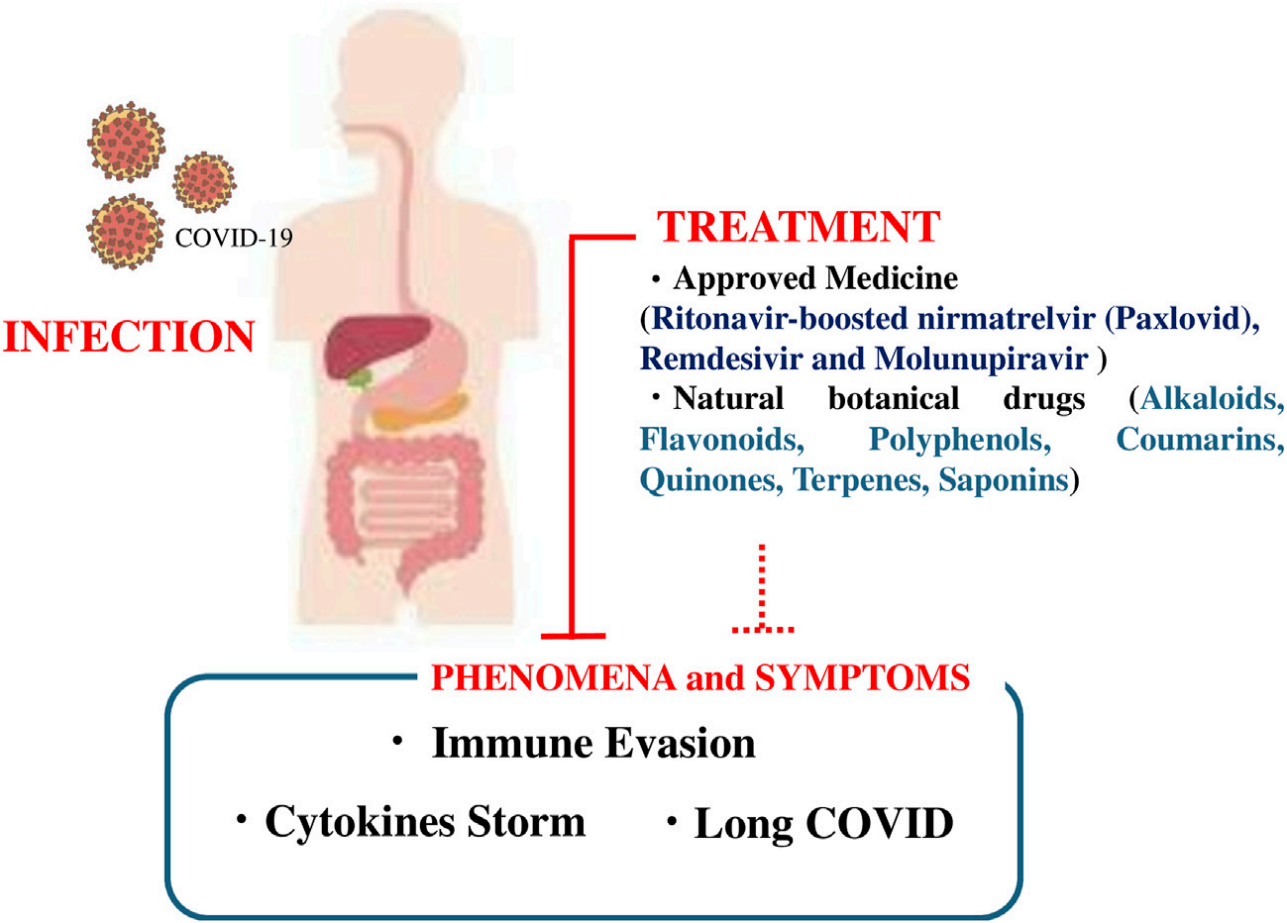
Schematic figure showing the infection process and symptoms of coronavirus disease 2019 (COVID-19), approved medicines (ritonavir-boosted nirmatrelvir [Paxlovid], remdesivir, and molnupiravir), and botanical drugs (alkaloids, flavonoids, polyphenols, coumarins, quinones, terpenes, and saponins).

We summarized the pre-existing comorbidities of patients with COVID-19, the prevalence of the most frequent underlying medical conditions in a sample of adults hospitalized with COVID-19, the leading causes of death in patients with COVID-19, the clinical symptoms, as well as available treatments, with a focus on established medical therapies and natural botanical drug-based treatments (summarized in Figure 3). Approximately 10% of patients with COVID-19 are affected by long COVID, and SARS-CoV-2 infection impairs mitochondrial DNA. Drugs approved for the treatment of COVID-19, such as ritonavir-boosted nirmatrelvir, remdesivir, and molnupiravir, have adverse effects. Since the number of pharmacological agents to treat COVID-19 is limited, adjuvant botanical drugs, including vitamin C and E supplementation, can reduce symptoms and may inhibit the progression to long COVID.
